# Innovative professional training approaches on the german national clinical guideline for NSSI in adolescents

**DOI:** 10.1192/j.eurpsy.2021.614

**Published:** 2021-08-13

**Authors:** E. König, U. Hoffmann, P. Plener, J. Fegert

**Affiliations:** 1 Child And Adolescent Psychiatry/psychotherapy, University Hospital Ulm, Ulm, Germany; 2 Child And Adolescent Psychiatry, Medical University of Vienna, Vienna, Austria

**Keywords:** non-suicidal self injury, E-Learning

## Abstract

**Introduction:**

German mental health care providers report to encounter many cases of NSSI in their professional context, but only around 50% know about the treatment guidelines for NSSI of children and adolescents. Many consider professional training about NSSI as necessary for themselves. In response to this need, within the project Star Train different strategies of dissemination of the content of the guidelines were developed.

**Objectives:**

This study aims at comparing the effectiveness of different strategies of dissemination: printed material, e-learning and blended-learning.

**Methods:**

Participants were randomly assigned to one of three different learning formats: printed material, e-learning, and blended-learning. Via online-survey participants provide pre- and post-training self-assessments of their knowledge of NSSI, practical skills, self-efficacy in handling cases of NSSI and attitudes towards NSSI and those affected. Additionally a multiple-choice-test of NSSI is assessed. For data-analyses between-group and within-group comparisons were conducted using a mixed design ANOVA. To ensure quality of learning formats, user-satisfaction was surveyed.

**Results:**

Data of the pretest-posttest comparison group design show that knowledge, practical skills, and self-efficacy in handling cases of NSSI raise for all participants and that attitudes towards NSSI and those affected are improved. There is no difference between the learning formats, thus all participants profit equally from their education. User satisfaction is high.

**Conclusions:**

Results of this study suggest that the developed different training strategies can contribute equally to a better understanding and enhance skills of professionals regarding NSSI. The choice of training method could be driven by considering which goals want to be achieved and cost-value ratio.

